# Quantification of dopaminergic neuron differentiation and neurotoxicity via a genetic reporter

**DOI:** 10.1038/srep25181

**Published:** 2016-04-28

**Authors:** Jun Cui, Megan Rothstein, Theo Bennett, Pengbo Zhang, Ninuo Xia, Renee A. Reijo Pera

**Affiliations:** 1 Department of Cell Biology and Neurosciences, Montana State University, Bozeman, MT 59717, USA; 2 Department of Pathology, Stanford University School of Medicine, Stanford, CA 94305, USA; 3 Department of Chemistry and Biochemistry, Montana State University, Bozeman, MT 59717, USA

## Abstract

Human pluripotent stem cells provide a powerful human-genome based system for modeling human diseases *in vitro* and for potentially identifying novel treatments. Directed differentiation of pluripotent stem cells produces many specific cell types including dopaminergic neurons. Here, we generated a genetic reporter assay in pluripotent stem cells using newly-developed genome editing technologies in order to monitor differentiation efficiency and compare dopaminergic neuron survival under different conditions. We show that insertion of a luciferase reporter gene into the endogenous tyrosine hydroxylase (TH) locus enables rapid and easy quantification of dopaminergic neurons in cell culture throughout the entire differentiation process. Moreover, we demonstrate that the cellular assay is effective in assessing neuron response to different cytotoxic chemicals and is able to be scaled for high throughput applications. These results suggest that stem cell-derived terminal cell types can provide an alternative to traditional immortal cell lines or primary cells as a quantitative cellular model for toxin evaluation and drug discovery.

One hindrance to research on human disease is inaccessibility of disease-relevant human cells. This has implications for understanding human disease; for example, despite intensive study over the years, mechanisms of neurodegenerative disorders such as the Parkinson’s Disease (PD) are still not completely understood. However, recent advances in human embryonic stem cells (hESCs)^1^ and induced pluripotent stem cells (iPSCs)^2^,^3^ may provide a reliable source of human cells. With directed differentiation, these pluripotent cells can be differentiated into diverse cell types, including dopaminergic (DA) neurons that are relevant to our understanding of PD and thus may provide new opportunities for disease modeling^4^,^5^,^6^. A number of related protocols have been developed to differentiate pluripotent stem cells into functional DA neurons that can mimic PD symptoms in humans and animal models^7^,^8^,^9^,^10^,^11^,^12^,^13^. Using stem cell-derived terminal cell types as a cellular disease model has advantages over conventional cell-based assays with immortalized cell lines or frozen human tissues as they provide a dynamic developmental system from birth to death of differentiated cells in cellular environments that physiologically mimic developmental processes^14^.

In order to model mechanisms of PD with pluripotent stem cells, a method to quantify DA neurons in differentiating cultures is important. In current differentiation methods, the differentiation process generally requires approximately 2–4 weeks from starting stem cells to functional DA neurons. Moreover, the efficiency of generating DA neurons varies significantly among different methods and may be affected by different cellular and environmental factors. Usually, approximately 20–30% of the final cells are DA neurons even with the most robust method such as the floor-plate induction protocol^13^. In this study, we developed a genetic reporter and used it to monitor the growth of stem cell-derived DA neurons during differentiation. Recent genome editing technologies such as Transcription Activator-Like Effector Nuclease (TALEN) technology^15^,^16^,^17^,^18^ provide an easy tool to directly edit target DNA sequences in the cell genome to fit specific experimental needs. With this technology, we engineered an hESC line by knocking in a secreted *Metridia* luciferase (Mluc) reporter gene^19^ in the endogenous Tyrosine Hydroxylase (TH) locus in hESCs. The reporter gene was then compared to that of endogenous expression of the TH gene during the process of differentiation of DA neurons. Because of the secreted nature of the reporter molecule, direct differentiation of the DA neural lineage was monitored non-invasively in real time for as long as 6 weeks in 96 and 384-well culture formats. We suggest that this strategy, of using a genetic reporter, provides a robust and specific measurement of target cell types and is suitable to be used in large scale quantitative experiments and screening assays.

## Results

### Generation of hESCs carrying the knock-in reporter

**Figure 1:**
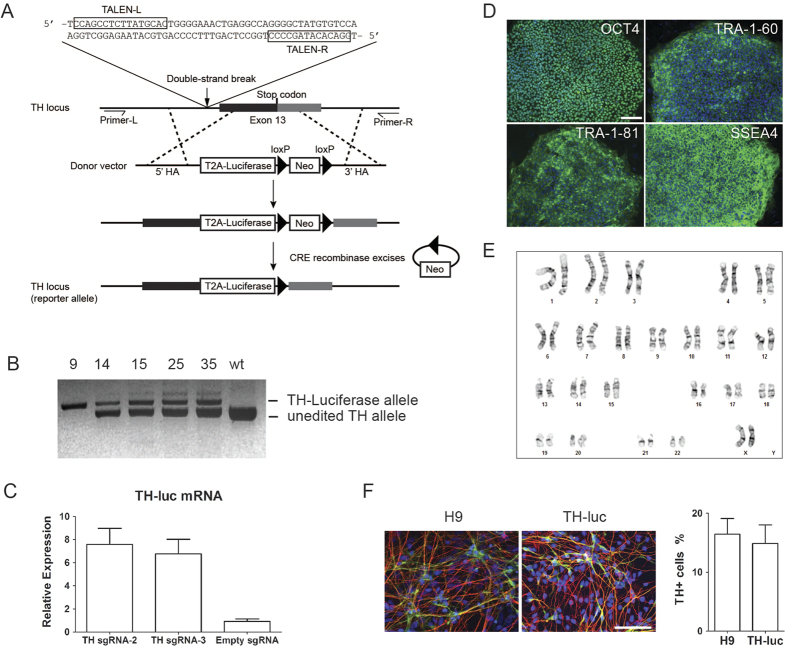
Creation of the TH knock-in reporter. (**A**) Schematic of genomic editing of the TH locus. A donor cassette carrying the T2A sequence, luciferase gene and neomycin selection gene was inserted into the locus through double strand break mediated homologous recombination. A transient CRE expression was then used to excise the drug selection gene to create the final reporter allele. (**B**) Genotyping of the modified clones using junction PCR with primers L and R as shown in (**A**). (**C**) CRISPR-mediated activation of TH transcription. Three independent samples were used for each vector. (**D**) Immunofluorescence staining of reporter ES cells for pluripotent markers OCT4, TRA-1-60, TRA-1-81 and SSEA4 (all in green). Nuclei were counterstained with DAPI (blue). (**E**) Chromosome analysis with G-banded karyotyping after genome editing. (**F**) Coimmunofluorescence staining of neurons derived from reporter ES cells after 5 weeks of differentiation for neuron-specific TUBB3 (red) and TH (green). Nuclei were counterstained with DAPI (blue). Scale bars = 100 μm.

To genetically label dopaminergic neurons, we chose to modify the TH gene which encodes the rate-limiting enzyme responsible for conversion of the amino acid L-tyrosine to the dopamine precursor L-3,4-dihydroxyphenylalanine (L-DOPA) in dopaminergic neurons. We genetically-modified the endogenous TH locus in the hESC line, H9 (WA09), using a two-step genome editing strategy as outlined in Fig. 1A. A pair of TALENs that specifically recognizes the intronic sequence near the editing site was used to achieve high homologous recombination efficiency of the region^18^. With a donor cassette, the endogenous stop codon of TH was deleted and the Mluc coding sequence was inserted downstream. To minimize effects on expression and translation of endogenous TH, a T2A sequence^20^ was placed in frame between the two coding regions to result in transcription of a bicistronic transcript that would be translated into two separate peptides. A floxed neomycin selection cassette was also included for selecting positive clones from homologous recombination and then excised from the genome by transiently expressing the Cre recombinase in the edited cells.

We recovered five engineered hESC clones after the genome editing procedure. Junction PCR amplifying the engineered genomic region was performed to genotype the clones (Fig. 1B). The parental H9 cells carry only the unmodified TH allele, while the modified allele is about 800 base pairs larger than wild-type in molecular size because of the insertion of the reporter gene. One of the clones, clone 9, only yielded the modified band while the other clones yielded both modified and wild-type bands, suggesting in clone 9 both TH alleles were modified to carry the reporter gene and all others have just one TH allele modified.

To test whether the modified reporter allele is regulated similar to the endogenous TH, we ectopically activated the transcription of the TH gene locus in the reporter cells with the CRISPR (clustered regularly interspaced short palindromic repeats) activator^21^,^22^. Small guide RNAs (sgRNAs) were designed to bind to the endogenous TH promoter and recruit a catalytically inactive Cas9 (dCas9) fused to a transcription activator to turn on transcription. TH-luciferase mRNAs were detected in the presence of the TH specific sgRNAs but not with the control scramble sgRNA (Fig. 1C). This result suggests that the transcription of the reporter can be activated by the endogenous TH regulation machinery.

Engineered cells exhibited morphology similar to the parental hESCs in that they were immunoreactive for pluripotency markers including OCT4, SSEA4, TRA-1-60, and TRA-1-81 (Fig. 1D), and maintained the normal karyotype (Fig. 1E). We then differentiated the reporter cells to DA neurons using an established floor-plate induction protocol^13^,^23^. The engineered cells can be differentiated robustly into the neural lineages similar to the parental H9 cells as indicated by the neural mark beta III tubulin and formed TH+ neurons after 5 weeks of differentiation (Fig. 1F). The above results combined indicate the reporter hESCs maintain pluripotency and the similar differentiation potential towards TH neurons to the parental H9 cells.

### Quantification of DA neurons during directed differentiation

**Figure 2:**
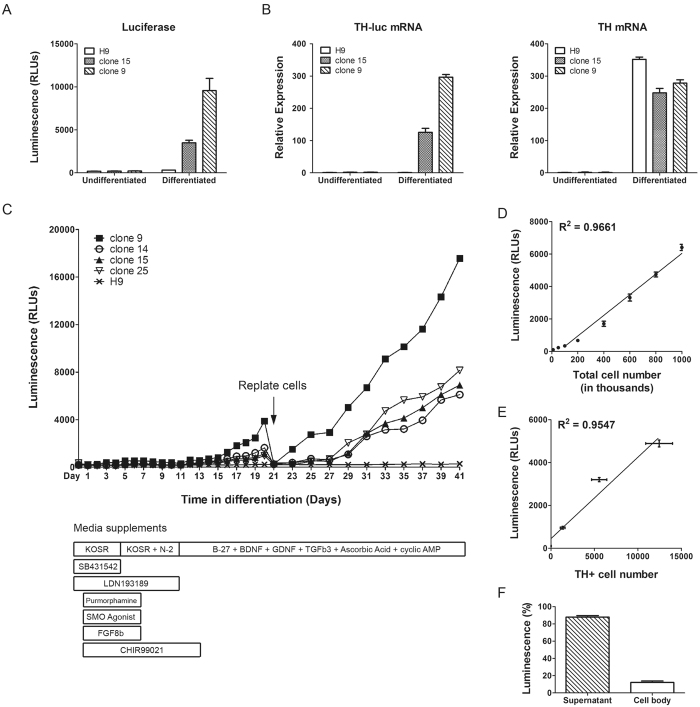
Quantification of DA neuron differentiation with the luciferase reporter. (**A**) Luciferase assay was performed in the cell culture media from ES cells (undifferentiated) or DA neurons derived from ES cells after 35 days of culturing (differentiated). Three independent samples were used for each line. (**B**) Quantitative PCR results of TH-luciferase hybrid transcripts or total TH transcripts. Expression was normalized using the housekeeping gene GAPDH and represented as folder change compared with undifferentiated ES cells. Three biological replicates were used. (**C**) Time course of a 6-week differentiation experiment. ES cells were differentiated with defined growth supplements as shown. After 20 days of differentiation, cells were split at 1:10 ratio onto new plates. Cell culture media were collected every day for the first 21 days and every other day of the following days. Three independent time course experiments were performed and the figure shows the result from one typical experiment. (**D**) Linear correlation of luciferase activity in DA neural culture with total cell numbers. Differentiated neurons were plated and recovered for 4 days before media were collected for the luciferase assay. Six wells were used for each cell number in each experiment and two independent experiments were performed. (**E**) Linear correlation of luciferase activity in DA neural culture with TH+ cell numbers. Luciferase assay was first performed and TH+ cells were then counted after immunofluorescent staining of the same well. Three wells were used for each cell number in each experiment and two independent experiments were performed. (**F**) Cellular partitioning of luciferase in DA neural culture. Media and cell lysates were separated and tested for luciferase activity. Six wells from three independent experiments were used for each sample in luciferase assays. Error bars represent standard error of mean in all panels.

Next, we tested whether expression of the luciferase reporter gene can be used to quantify neural differentiation. ES cells were induced to develop to DA neurons following the floor-plate protocol and luciferase assay was performed to test the luciferase activity in cell culture media (Fig. 2A). Luciferase activity was detected in DA neurons derived from reporter clones 9 and 15 but not from wildtype (un-engineered) H9 parental cells or undifferentiated ESCs. Note that the neural culture of clone 9 produced luciferase signal roughly twice of that from clone 15. This result is consistent with the previous finding that clone 9 carries two copies of the reporter gene. We also performed a quantitative PCR assay that detects the TH-luciferase hybrid mRNA with one primer binds the coding region of TH and the other binds luciferase. The hybrid mRNA expression was detected in DA neurons from reporter clones 9 and 15 but not from H9 cells (Fig. 2B), which is consistent with the luciferase activity.

We next conducted a 6-week time course experiment to examine the TH expression pattern during direct DA differentiation using our reporter assay (Fig. 2C). Expression of TH was not detected in the first 10 days of differentiation when the stem cells were induced to adopt a floor-plate fate by a pre-defined combination of growth factors. On Day 11, the culture media were switched to the neural growth condition containing B-27 supplements, Brain-derived neurotrophic factor (BDNF), Glial cell-derived neurotrophic factor (GDNF), Transforming growth factor beta 3 (TGFb3), ascorbic acid and cyclic AMP (cAMP). Expression of TH was detected starting from Day 11 and continued to increase throughout the following culturing in the neural growth media. It has been established that TH expression in neurons is induced and maintained in the presence of cAMP^24^. This result suggests that TH+ neurons start to form after Day 11 and continue to grow throughout the remaining of the differentiation. Signal intensity of the luciferase was linearly correlated with the total cell number seeded (Fig. 2D) and the number of TH positive cells (Fig. 2E). Moreover, the majority of the luciferase (>90%) expressed was secreted into the supernatant of the culture medium, thus enabling simple and instantaneous measurements via use of the supernatant of the cell culture (Fig. 2F). Note, that in our experience, and as expected, single allele genetic modification (knock-in) is more frequent than modification of both alleles simultaneously. Thus, we used the heterozygous clone 15 in subsequent experiments, unless otherwise indicated, to probe performance of genetic labeling with one copy of a genetic reporter.

### Differentiation efficiency and cell densities

**Figure 3:**
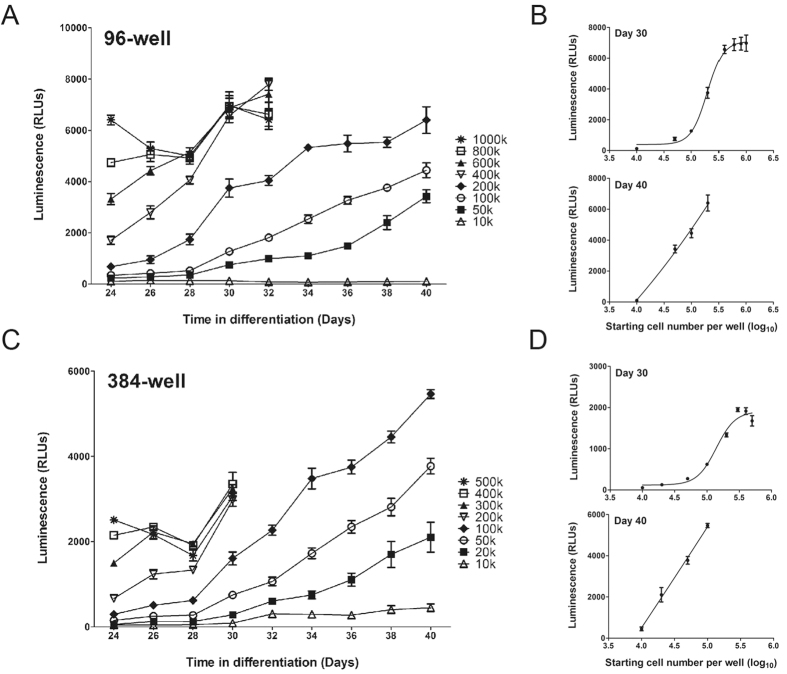
Differentiation outcome with different cell densities. Early neural cells were detached and separated into single cells on Day 20 of differentiation and replated onto microwell plates at a series of cell densities shown in the figure. DA neuron growth was examined using the luciferase assay every two days with cells grown in 96-well format (**A**) or 384-well format (**C**). Differentiation of the four high density wells were terminated on Day 32 due to over-growth and massive cell detachment. Luciferase signal intensity was plotted with the cell number on Days 30 and 40 for cells grown in 96-well format (**B**) or 384-well format (**D**). Six wells were used for each sample in each experiment. Three independent time course experiments were performed and the figure shows the result of one typical experiment. Error bars represent standard error of mean in all panels.

One parameter that directly affects the yield of DA neurons in the final culture is cell density. Replating cells at a higher density can optimize DA neuron development and increase the final yields^13^. To identify the optimal cell density for DA neuron differentiation and test performance of our reporter assay, on Day 20 we replated differentiated neural cells at different densities and followed the DA neuron growth for two weeks. As shown in Fig. 3A, the optimal starting cell density in 96-well plates ranged from 50 K to 200 K cells per well, or 150 K to 600 K cells per cm^2^ considering the surface area of one well as 0.33 cm^2^. When cells were plated at densities higher than 400 K cells per well, restraint by the surface growth area greatly limited neuron attachment and growth resulting in massive cell detachment within several days. While fewer than 50 K cells per well led to very poor cell attachment and a low total number of DA neurons. Within the optimal range, each well produced 10^3^ to 10^4^ units of luminescence signal when the culture matured (Fig. 3B). The signals showed a linear correlation with cell numbers on Day 40, suggesting the cell growth was not restricted during the culturing period. The growth test was also performed in the 384-well format and exhibited a similar result with optimal cell densities of 20 K to 100 K cells per well (Fig. 3C,D). These results indicate that the reporter is suitable for used as luciferase based quantitative assays.

### Neurotoxicology of oxidative stressors on DA neurons

**Figure 4:**
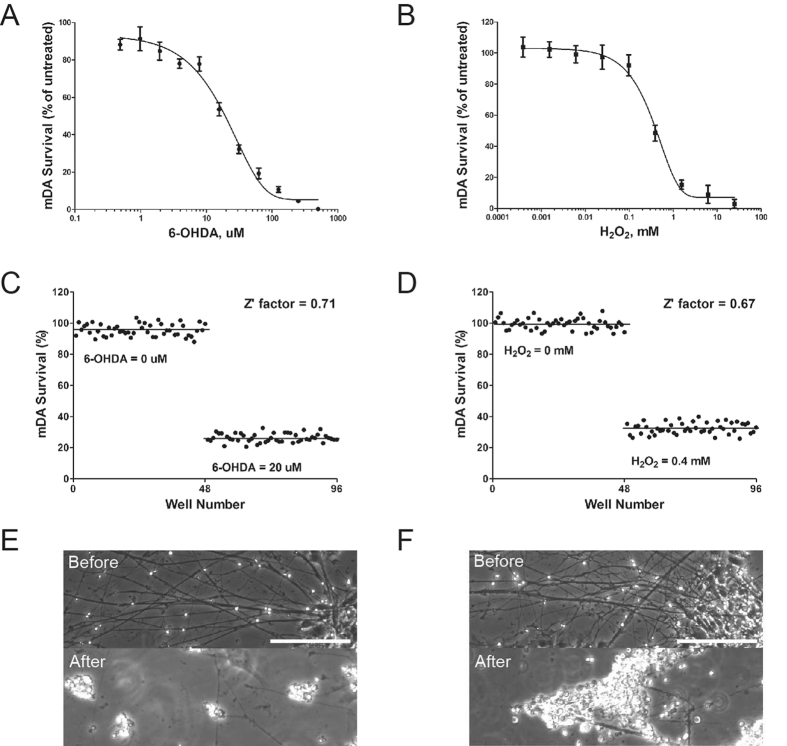
Chemical-induced neurotoxicity in differentiated DA neurons. Concentration-response curves for oxidant stressors 6-OHDA (**A**) and H_2_O_2_ (**B**). Neurons differentiated from ES cells for 5 weeks were exposed to the chemical at final concentrations shown in the figure for 48 hours. Survival of TH+ neurons was calculated as the percentage of luminescence signal after treatment vs. before treatment of the same well. Four wells were used at each concentration and the experiments were performed three times. Error bars represent standard error of mean in all panels. (**C**,**D**) Z′-factor was calculated using 48 wells in each group. Neurons were degenerated after treatment with 20 uM 6-OHDA (**E**) and 0.4 mM H_2_O_2_ (**F**). Scale bars = 100 μm.

PD-associated mutants have linked the loss of DA neurons to mitochondrial dysfunction and impaired mitochondrial function is a possible cause of increased oxidative stress in the cells^25^. In previous studies we found that DA neurons were especially vulnerable to oxidative stress induced by toxins such as H_2_O_2_ and 6-hydroxydopamine (6-OHDA)^12^,^26^ and studying DA neuron response under oxidative stress could be a useful *in vitro* model for PD. In these studies, DA neurons were quantified with immunostaining and counting TH immunostained neurons which could be costly and slow. We tested if our reporter assay can be used for neurotoxicity studies. We derived reporter ES cells to DA neurons as described above and then exposed them to different concentrations of the neurotoxins. Survival rates of TH+ neurons were calculated based on the luminescent signal from the same well before and after chemical treatment to generate the concentration-response relationships of the TH+ neurons responding to these chemicals (Fig. 4). We determined that the LC50 (lethal concentration 50) of 6-OHDA is 15.2 uM and 0.306 mM for H_2_O_2_ treatment of TH+ neurons. Furthermore our cell-based assay showed excellent Z′ scores at 0.71 and 0.67 after 6-OHDA (Fig. 4C) or H_2_O_2_ (Fig. 4D) treatment respectively, suggesting that the assays are suitable for use in high throughput screening applications.

## Discussion

Generation of lineage specific knock-in reporter pluripotent stem cell lines can provide an effective strategy for tracking gene expression during lineage differentiation. Most applications in other studies use a fluorescent protein reporter system^27^,^28^,^29^. In a separate study, we have developed a fluorescent reporter for TH using the similar strategy and successfully isolated purified TH neurons during differentiation (data not shown). However, it is clear that fluorescent reporters are limited predominantly to end-point assays rather than as a monitoring tool for real time assessement of living cells; this is largely due to weak fluorescence signals produced from the TH gene locus. This is likely a common problem with diverse lineage-specific reporters as endogenous expression of most lineage specific marker genes may be low. Here, we used a luminescent reporter system to overcome this limitation and provide convenient and quantitative measurements instantaneously. Using this assay, we monitored TH expression over long-term differentiation and examined parameters of cell culturing that may achieve higher differentiation efficiencies. The floor-plate induction method that we used produced DA neurons in two stages: A TH negative induction stage and a TH positive neural growth stage which correlate with the media condition. The reporter system described here provided continuous measurements over the transition from one stage to the next.

In this study, we directly engineered the endogenous TH gene locus in the cell genome. Our strategy is different from the commonly used conventional luciferase assay in which the reporter gene is hooked with a lineage specific promoter and brought into the cells often using a DNA plasmid or a viral vector which might integrate into the cell genome randomly. Because the integration site is unpredictable, it could sometime result in a non-specific background expression of the reporter gene in 1–5% cells (data not shown). This background noise cannot be overlooked considering that only ~20% of the differentiated cells are real TH positive neurons in the culture. Insertion of the reporter directly in the same gene locus is a more controllable approach and can place the reporter under the same endogenous gene expression regulation of the target gene that minimizes the background expression.

The data provided here also demonstrate that stem cell-derived DA neurons provide a feasible cellular model for neurotoxicology studies. Because the neurons can be produced from human pluripotent stem cells almost unlimitedly at a relatively low cost, it has a great potential to be further developed as a high throughput screening method for discovering new reagents. Genetically labeling a lineage specific gene in pluripotent stem cells combined with a robust lineage direct differentiation protocol enables one to easily build *in vitro* quantitative assays for specific terminal cell types. The system outlined provides a cost-effective and scalable alternative relative to use of primary cells or animal models. We suggest that stem cell derived functional cells have the potential to contribute to a highly specific and robust *in vitro* cell-based platform for the purposes of drug testing and screening studies.

## Methods

### Human embryonic stem cell culture

Human ES cell line H9 was obtained from WiCell Research Institute and maintained on Matrigel (BD Biosciences) in Essential 8 medium (Life Technologies). Every 3–5 days ES cells were detached with Collagenase (Life Technologies) and passaged at 1:10 split to new plates freshly coated with Matrigel.

### Genome editing of the endogenous TH gene locus

TALENs specific for the human TH gene were designed and constructed following the method described previously^30^,^31^. One TALEN pair that recognizes the intronic region near TH’s endogenous stop codon was used in the following gene editing experiment. A homologous recombination donor construct was assembled to carry the five components in the following order: a 1 kb 5′ homologous arm, T2A sequence, *Metridia* luciferase coding sequence, a neomycin selection cassette flanked by loxP sites and a 1 kb 3′ homologous arm. For transfection, 2 × 10^6^ ES cells were transfected with 10 μg of the donor plasmid and 1 ug of each TALEN-coding plasmid using Amaxa Nucleofector (Lonza) following the manufacturer’s instruction. 10 uM Y-27632 (Sigma) was added to the medium during the process to improve the survival rate of ES cells. Cells were selected in medium containing 50 ug/ml geneticin for 2 weeks and survival colonies were manually picked and expanded. Genomic DNA was extracted from each clone using Quick-gDNA miniprep kit (Zymo Research) and screened using junction PCR. Cells with integration of the donor cassette in the target locus were then transfected with a plasmid expressing the CRE recombinase to excise the neomycin gene. Final clones were genotyped by junction PCR and further confirmed with Sanger sequencing the PCR products.

### CRISPR activation of transcription

A catalytically inactive Cas9 (dCas9) fused to a transcription activator was expressed using the lentiviral construct pHAGE EF1a dCas9-VP64 (Addgene plasmid 50918). The original construct was modified by replacing the PGK promoter of the puromycin resistant gene with a 2A peptide so that dCas9-VP64 and Puro can be transcribed bi-cistronically. The modified lentiviral construct was packaged into viral particles and used to infect TH-luciferase reporter hESCs. Stable clones were established and maintained in culture in the presence of puromycin selection. Small guide RNAs specific for the TH promoter were designed and synthesized following the published protocol^32^. Three days post transfection of sgRNAs, cells were collected for RNA extraction and qPCR was then performed to examine TH-luciferase hybrid mRNA.

### RNA extraction and quantitative PCR analysis

Total RNA was extracted using RNeasy mini kit (Qiagen). For first-strand cDNA synthesis, 2 μg of total RNA was reverse transcribed with oligo-dT primers and SuperScript III reverse transcriptase (Life Technologies). Quantitative PCR reactions were run using Power SYBR PCR mix (Life Technologies) and detected by ABI 7300 Real-Time PCR System. Relative quantity for each sample and gene was normalized and calculated based on the ΔCt values using GAPDH as the control.

### Immunofluorescence staining

Cells were fixed with 4% paraformaldehyde/PBS for 30 minutes at room temperature. Fixed cells were washed two times with PBS/0.1% Tween-20 and blocked for 30 minutes in PBS containing 4% normal goat serum at room temperature. Cells were incubated at 4 °C overnight with primary antibodies. Subsequently, cells were washed three times with PBS/0.1% Tween-20 and incubated with fluorescent-conjugated secondary antibodies for 1 hour at room temperature. Nuclei DNA were stained with 1 ug/ml DAPI. Stained cells were imaged on a Leica inverted microscope. Primary antibodies were diluted as follows: Oct4 (Santa Cruz) at 1:100, Sox2 (Millipore) at 1:200, TRA-1-60 (Millipore) at 1:200, TRA-1-81 (Millipore) at 1:200, TH (Pel Freez) at 1:500, TUJ (Abcam) at 1:1000.

### Directed dopaminergic differentiation and quantification of TH using luciferase assay

ES cells were differentiated towards a dopamingeric neuron cell fate for up to 6 weeks following the floor-plate induction method^23^. Cell supernatants were collected every day for the first 20 days of neuronal differentiation and every other day afterwards. Luminescent reactions were performed by mixing the supernatants with 10× reaction buffer consist of 300 mM Tris-HCl pH 8.0, 40 mM NaCl, 1% Triton X-100 and 200 μM substrate Coelenterazine (NanoLight). Luminescent signal was measured using a FlexStation 3 Microplate Reader (Molecular Devices). Background signal level was measured using blank medium with reaction buffer and subtracted from experimental data.

### Neurotoxicity assays

ES cells were differentiated towards dopamingeric neurons for 20 days as described above. On Day 20, neurons were dissociated by incubating with Accutase (Innovative Cell Technologies) for 5 minutes. Dissociated cells were counted and replated to 96-well plates coated with Poly-L-ornithine/Laminin/Fibronectin at a density of 3 × 10^5^ cells/cm^2^ and allowed to recover for one week in Neurobasal medium containing B-27 supplement, BDNF, GDNF, cAMP, TGFb, and Ascorbic Acid. Neurotoxin treatment was performed as previously described^12^. Day 30 to 40 derived neurons were used in all the assays. Fleshly prepared H_2_O_2_ solution (30%, Fluka) or 6-hydroxydopamine (Sigma) was diluted with the basal medium and added to each well to reach the desired final concentration. Assays were done with multiple wells of 4 replicates for each condition. Whole supernatants of the same well were collected before drug treatment and 48 hours post treatment for quantification using luciferase assay as described above. Viability of TH+ cells was calculated as the percentage of luminescent signal intensity post treatment compared to that before treatment of the same well.

## Additional Information

**How to cite this article**: Cui, J. *et al.* Quantification of dopaminergic neuron differentiation and neurotoxicity via a genetic reporter. *Sci. Rep.* **6**, 25181; doi: 10.1038/srep25181 (2016).
